# Microvesicles from human adipose stem cells promote wound healing by optimizing cellular functions via AKT and ERK signaling pathways

**DOI:** 10.1186/s13287-019-1152-x

**Published:** 2019-01-31

**Authors:** Sen Ren, Jing Chen, Dominik Duscher, Yutian Liu, Guojun Guo, Yu Kang, Hewei Xiong, Peng Zhan, Yang Wang, Cheng Wang, Hans-Günther Machens, Zhenbing Chen

**Affiliations:** 1 Department of Hand Surgery, Union Hospital, Tongji Medical College, Huazhong University of Science and Technology, No. 1277 Jiefang Avenue, Wuhan, 430022 China; 2 Department of Plastic and Hand Surgery, Technical University of Munich, Ismaninger Strasse 22, 81675 Munich, Germany

**Keywords:** Adipose stem cells (ASCs), Microvesicles (MVs), Extracellular vesicles, Wound healing

## Abstract

**Background:**

Human adipose stem cells (ASCs) have emerged as a promising treatment paradigm for skin wounds. Recent works demonstrate that the therapeutic effect of stem cells is partially mediated by extracellular vesicles, which comprise exosomes and microvesicles. In this study, we investigate the regenerative effects of isolated microvesicles from ASCs and evaluate the mechanisms how ASC microvesicles promote wound healing.

**Methods:**

Adipose stem cell-derived microvesicles (ASC-MVs) were isolated by differential ultracentrifugation, stained by PKH26, and characterized by electron microscopy and dynamic light scattering (DLS). We examined ASC-MV effects on proliferation, migration, and angiogenesis of keratinocytes, fibroblasts, and endothelial cells both in vitro and in vivo. Next, we explored the underlying mechanisms by gene expression analysis and the activation levels of AKT and ERK signaling pathways in all three kinds of cells after ASC-MV stimulation. We then assessed the effect of ASC-MVs on collagen deposition, neovascularization, and re-epithelialization in an in vivo skin injury model.

**Results:**

ASC-MVs could be readily internalized by human umbilical vein endothelial cells (HUVECs), HaCAT, and fibroblasts and significantly promoted the proliferation, migration, and angiogenesis of these cells both in vitro and in vivo. The gene expression of proliferative markers (cyclin D1, cyclin D2, cyclin A1, cyclin A2) and growth factors (VEGFA, PDGFA, EGF, FGF2) was significantly upregulated after ASC-MV treatment. Importantly, ASC-MVs stimulated the activation of AKT and ERK signaling pathways in those cells. The local injection of ASC-MVs at wound sites significantly increased the re-epithelialization, collagen deposition, and neovascularization and led to accelerated wound closure.

**Conclusions:**

Our data suggest that ASC-MVs can stimulate HUVEC, HaCAT, and fibroblast functions. ASC-MV therapy significantly accelerates wound healing, and the benefits of ASC-MVs may due to the involvement of AKT and ERK signaling pathways. This illustrates the therapeutic potential of ASC-MVs which may become a novel treatment paradigm for cutaneous wound healing.

**Electronic supplementary material:**

The online version of this article (10.1186/s13287-019-1152-x) contains supplementary material, which is available to authorized users.

## Background

Acute and chronic skin injuries, resulting from burns, pressure, diabetes mellitus, and venous stasis, pose a great burden on society [1, 2]. Normal wound healing is one of the most complicated biological processes. It requires the accurate cooperation of many types of cells and the precise coordination of various biological and molecular events [3]. Despite of huge investments in this field, progress has been limited, especially in the treatment of chronic wounds.

Stem cell-based therapies open a new door for tissue repair and have been widely studied in the context of regeneration medicine. The application of mesenchymal stem cells (MSCs), embryonic stem cells, or pluripotent stem cells (PSCs) for the treatment of skin wounds has shown beneficial outcomes in preclinical and early clinical studies [4]. Adipose stem cells, as a member of mesenchymal stem cells, have been recognized as one of the most promising stem cell sources because of a series of advantages. The harvest of adipose tissue is easy and often accompanied by esthetic benefits. Additionally, the frequency of stem cells in adipose tissue is high and more abundant than other origins such as bone marrow [5]. Adipose stem cells (ASCs) have been well-documented to have therapeutic effects on skin wound healing, cardiac injury, immune disorders, and other indications of ischemia and tissue loss [6–8]. Although great advances have been made in animal models, the clinical translation of ASC-based therapies has been problematic [9]. Cell therapeutic approaches are accompanied by significant regulatory hurdles and require delicate handling at all steps of harvesting, processing, and transplantation [10]. Transplanted cells have only limited viability in the harsh wound environment [11]. Additionally, there is little evidence of the differentiation of stem cells into the specific type of cells for replacing the cells at the site of injury [12]. Importantly, recent works have demonstrated that paracrine factors significantly contribute to the therapeutic effect of stem cells on tissue repair, and extracellular vesicles may play an important role [13]. Harnessing their regenerative potential could overcome many translational hurdles of cellular therapy.

Extracellular vesicles comprising exosomes and microvesicles are perceived as mediators for intercellular communication, allowing the exchange of DNA, RNA, proteins, and lipids between cells [14]. Exosomes are referred to small membrane vesicles (50–150 nm in diameter), whose secretion requires the fusion of multivesicular endosomes with the plasma membrane. The effect of exosomes from various types of cells on tissue repair has been widely evaluated in recent studies. Previous works showed that exosomes from fibrocytes, endothelial progenitor cells (EPCs), human induced pluripotent stem cell-derived MSCs (hiPSC-MSCs), and human umbilical cord MSCs (hucMSCs) could promote wound healing by modulating cellular function and enhancing angiogenesis [15–18]. Additionally, exosomes from ASCs have also been demonstrated to accelerate wound healing via optimizing fibroblasts function [19, 20]. In comparison, microvesicles are large membrane vesicles (range 50–1000 nm in diameter) and generate from the plasma membrane by outward budding. Microvesicles from myofibroblasts and platelets have shown a therapeutic effect on wound healing by promoting angiogenesis of endothelial cells [21, 22]. The difference of physical function between exosomes and microvesicles has yet to be illustrated [23]. Also, the effect of microvesicles from mesenchymal stem cells on wound repair is less documented compared to that of exosomes. Thus, this study aims to shed light on the regenerative effects of microvesicles from ASCs in the context of cutaneous wound repair.

## Methods and materials

### Cell isolation and culture

ASCs were isolated from the subcutaneous fat from patients consented in accordance with the Ethics Committee at the Tongji Medical College of Huazhong University of Science and Technology. The fresh fat specimen was washed three times with PBS containing 1% penicillin/streptomycin and chopped by sterile operation scissors. The chopped tissue was digested with 0.2% collagenase type I (Sigma, USA) at 37 °C for 1.5 h and centrifuged at 1500 rpm for 10 min. The cell pellet was resuspended and filtered through a 70-μm filter (Corning, USA). After another centrifugation for 5 min, the cell pellet was resuspended in the culture medium consisting of Dulbecco’s modified Eagle’s medium (DMEM, Gibco, USA) high glucose, 10% fetal bovine serum (FBS, Serapro, USA), and 1% penicillin/streptomycin. The culture medium was replaced 48 h after seeding to remove nonadherent cells; thereafter, the medium was replenished every 2–3 days. The ASCs at passages 2–7 were used for the following experiments. HaCAT (#GDC106, CCTCC) and human umbilical vein endothelial cells (HUVECs) (#GDC166, CCTCC) were obtained from the China Center for Type Culture Collection (CCTCC, Wuhan, China) and cultured following the manufacturer’s instructions. Human foreskin fibroblasts were isolated using previously described protocols [19].

### ASC characterization

Flow cytometry was used to observe the phenotypes of ASCs. Passage 3 ASCs were trypsinized and incubated with anti-CD90-FITC, CD73-FITC, anti-CD44-PE, anti-CD34-PE, anti-anti-HLA-DR-PE (Biolegend, San Diego, CA, USA) for 1 h. After two washes with PBS, the fluorescence of ASCs was observed.

#### In vitro adipogenic differentiation

Passage 3 ASCs were seeded in standard 6-well tissue culture plates (1.5 × 10^5^ cells per well) and incubated for 3 weeks with the adipogenic differentiation medium (Cyagen Biosciences Inc., Guangzhou, China). The cells were then fixed in 10% formalin and stained with Oil Red O for 30 min.

#### In vitro osteogenic differentiation

Passage 3 ASCs were seeded in 6-well tissue culture plates (2 × 10^5^ cells per well), precoated with 0.1% gelatin, and incubated for 2 weeks with osteogenic (Cyagen Biosciences Inc., Guangzhou, China) differentiation medium. The cells were then fixed and stained with Alizarin Red for 30 min. After three times washes with PBS, the stained cells were detected with microscopy.

### ASC-MV isolation and characterization

Cell culture supernatants were collected every 72 h from cells starting at passage 2 until passage 7, when the cells became 80–90% confluent. The FBS used for culturing ASCs was ultracentrifugated at 120,000*g* overnight to remove contained extracellular vesicles. MVs were isolated using previous protocol [24]. The supernatants were initially centrifuged at 1000*g* for 10 min to remove dead cells and later centrifuged at 4000*g* for 30 min to remove cell debris. The supernatants were then concentrated using 100 KDa molecular weight Amicon®Ultra-15 Centrifugal Filter Devices (Millipore, USA) and centrifuged at 13,000*g* for 1 h to obtain MVs. The MVs were washed once with PBS to remove contaminating proteins and stored at − 80 °C for the next experiences. The qualification of ASC-MVs was performed by transmission electron microscope (Hitachi, Japan) and dynamic light scattering (Malvern Instruments Ltd., Worcestershire, UK), and the protein level was quantified with Pierce BCA Protein Assay Kit (Aspen, China) as the manufacturer’s instructions.

### ASC-MV labeling and internalization assay

ASC-MVs were incubated with red fluorescent dye (PKH26, Sigma, USA) for 4 min and treated with 0.5% BSA/PBS to neutralize redundant dye. Then, the labeled MVs were obtained after centrifuged again to remove contaminating dye. For internalization assay, cells were seeded in the 35-mm confocal dish at proper density and treated with 20 μg labeled MVs. After incubation for 24 h, cells were washed twice with PBS and fixed in 4% paraformaldehyde for 10 min; thereafter, the nucleic was stained with DAPI (Solarbio, Beijing, China) and the cytoskeleton was stained with FITC phalloidin (Yeasen Biotech Co., Shanghai, China) according to the manufacturer’s instructions. The MV uptake by cells was observed by using the laser scanning confocal microscope.

### Cell proliferation and migration

Cells were trypsinized and seeded in 96-well tissue culture plates. After overnight incubation, the cell culture medium was replaced and simultaneously added with 20 μg/ml ASC-MVs or PBS. The cell number was calculated by CCK8 kit (Dojindo, Shanghai, China) at days 0, 1, 2, and 3 as the manufacturer’s instructions.

The migration of cells was performed in a 24-well Transwell chamber (8.0 or 12 μm pore size, Corning, USA). In brief, cell culture medium (DMEM/F12 with 10% FBS) was added to the lower compartment. Cells in 200 μl DMEM/F12 (Hyclone, USA) were added to the upper compartment and simultaneously treated with 10 μg/ml ASC-MVs, 5 μg/ml ASC-MVs, or PBS. After incubation at 37 °C for 24 h, the chamber was removed and the cells that migrated to the bottom of the chamber were stained with crystal violet staining (Solarbio, Beijing, China) and counted manually under microscopy in each well. Data are expressed as an average number of cells per field that migrated through pores.

### In vitro tube formation assay

HUVECs (2 × 10^4^ cells per well) were seeded with 20 μg/ml ASC-MVs or PBS in 48-well culture plates that had been coated with 130 μl Matrigel Basement Membrane Matrix (BD Biosciences, CA, USA). Tube formation was detected under microscopy at 2 h, 4 h, and 8 h incubation. Results are represented as mean ± SEM in three independent experiments.

### qRT-PCR

Cells were seeded in 12-well culture plates, starved overnight, and then treated with 20 μg/ml ASC-MVs or PBS. After 12 h of incubation, total RNA from cells was isolated with TRIzol Reagent (TaKaRa, Dalian, China) and transcribed to cDNA using PrimeScript® RT reagent Kit with gDNA Eraser (#RR047A, TaKaRa). Real-time PCR was performed according to the manufacturer’s instructions (Applied Biosystems, Carlsbad, CA, USA) with SYBR® Premix Ex Taq II (TaKaRa, Dalian, China). The primer sequences for each gene are described in Additional file 1: Table S1. Expression of targeted gene was assessed using the 2^−ΔΔCt^ method and normalized to GAPDH.

### Signaling experiments in cells

Starved cells were trypsinized and distributed equally into two different tubes. The cells were then centrifuged at 300*g* for 5 min and resuspended in serum-free medium supplemented with 20 μg/ml ASC-MVs or PBS. After the indicated length of incubation, the cells were collected and lysed in RIPA lysis buffer (Beyotime Biotechnology, Shanghai, China) added with protease and phosphatase inhibitor (Roche, Switzerland).

### Western blot

Western blot was performed using previously described protocols. Briefly, equal amount of total protein (20–40 μg) was separated by SDS-PAGE (Beyotime Biotechnology, Shanghai, China), transferred into the PVDF membrane (Millipore, USA), and then incubated overnight with primary antibodies specific for cyclin D1 (#A11022, ABclonal, China), VEGFR2 (#A5609, ABclonal, China), fibronectin (#A12932, ABclonal, China), α-SMA (#A7248, ABclonal, China), VEGFA (#ab52917, Abcam), cyclin A2 (#ab181591, Abcam), PDGFR (#ab69506, Abcam), collagen I (#ab138492, Abcam), collagen III (#ab184993, Abcam), elastin (#ab213720, Abcam), GAPDH (#10494-1-AP, Proteintech, China), ERK (#4695, Cell Signaling Technology), p-ERK (#4370, Cell Signaling Technology), AKT (#4691, Cell Signaling Technology), and p-AKT (#4060, Cell Signaling Technology). After incubated with HRP-conjugated antibody (Aspen, China) for 1 h, the membrane was incubated with Immobilon ECL substrate kit (Millipore, USA) for 1 min and then exposed to X-ray film using BioSpectrum 600 Imaging System (UVP, CA, USA).

### Wound healing model

All animal experiments were approved by the Animal Care Committee of Tongji Medical College. Eight-week-old male BALB/c mice were randomized into two groups: 50 μl PBS group (*n* = 9/group) and 50 μl ASC-MVs group (*c* = 1.0 μg/μl). As previously described, a 7-mm full-thickness cutaneous wound was excised on the midline of the mouse back, with each wound edge sutured with silicone rings (*d* = 1.0 cm) in order to prevent wound contraction. For the treatments of wounds, the wounds were subcutaneously injected with PBS or ASC-MVs at five sites once after the wounds were created. Digital photographs were taken at days 0, 3, 7, 10, and 13, and the wound area was measured using the Image J software.

### In vivo tracking experiment

Eight-week-old male BALB/c mice were randomized into three groups: 50 μl PBS group (*n* = 3/group), 50 μl PKH26 group, and 50 μl PKH26-labeled ASC-MVs group (*c* = 1.0 μg/μl). The wounds on mice back (1 × 1 cm) were subcutaneously injected with PBS, PKH26, or PKH26-labeled ASC-MVs once after the wounds were created. Fluorescence images were taken at days 1, 3, 5, 7, 10, and 15 by the in vivo imaging system (Bruker, German). The images were analyzed using Bruker MI SE 7.2 software.

### Histological analysis

The wounds were harvested at day 13 after surgery and fixed with 4% paraformaldehyde. After being dehydrated with a series of graded ethanol, the tissues were then embedded in paraffin and cut into 8-μm-thick longitudinal sections. The sections were stained with hematoxylin and eosin (H&E) for histological analysis of wound repair. In addition, Masson staining was carried out to evaluate collagen accumulation.

### Immunohistochemistry analysis

The sections were incubated with PCNA, CD34, ki-67, and α-SMA antibody (Abcam, 1:200) overnight at 4 °C. After being washed three times with PBS, the sections were incubated with a second antibody (Aspen, China) for 1 h at room temperature. The image was taken by a microscope, and then analyzed by using ImageJ software.

### Statistics

All data were expressed as the mean ± SEM. Comparisons between the two groups were evaluated with the unpaired Student’s *t* test. For group > 2, one-way or two-way ANOVA with Bonferroni post hoc test was used. Statistical significance was set at *p* < 0.05. Statistical analysis was conducted using GraphPad Prism v 7.0 software.

## Results

### Identification of ASCs and ASC-MVs

With the extension of incubation time, ASCs were gradually becoming homogeneous, formed a monolayer of adherent cells, and exhibited a typical fibroblast-like morphology (Additional file 2: Figure S1b). ASCs at passage 3 were collected and characterized by flow cytometry. The results showed that ASCs were strongly positive for CD73 (92.4%), CD90 (99.1%), and CD44 (99.1%) but negative for CD34 (2.15%) and HLA-DR (2.63%) (Additional file 2: Figure S1a). To identify the multipotency of ASCs, we performed adipogenic and osteogenic differentiation assays. After incubation in adipogenic differentiation medium for 3 weeks, most cells demonstrated adipocytic features as stained with Oil Red O (Additional file 2: Figure S1c). Similarly, after incubation in osteogenic differentiation medium, most cells could differentiate into osteoblasts as evidenced by Alizarin Red staining (Additional file 2: Figure S1d).

**Fig. 1:**
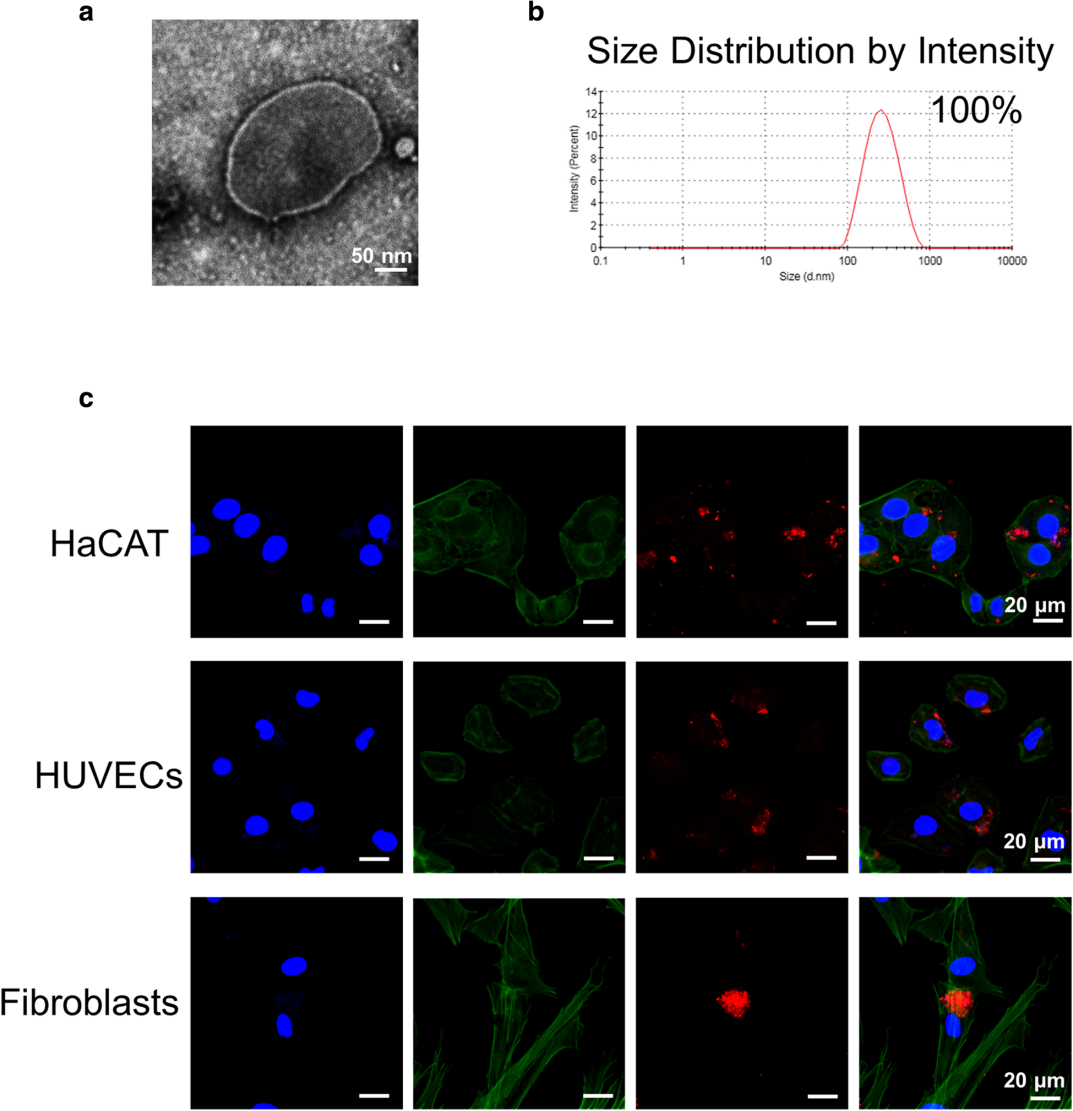
Isolation, characterization, and internalization of ASC-MVs. **a** Microvesicles isolated from ASC culture media were evidenced by electron microscopy. **b** Measurement of ASC-MV population by dynamic light scattering (DLS) demonstrated a single-peaked pattern (90–900 nm in diameter), which indicates isolated MVs were free of contamination. **c** Confocal images of HaCAT, HUVECs, and human foreskin fibroblasts incubated with either PBS or 20 μg PKH26-labeled MVs for 24 h

ASC-MVs were isolated by differential ultracentrifugation. Based on protein content, the average production of MVs was approximately 3 μg per million cells. Electron microscopy revealed that MVs were primarily circular and double membrane wrapped in shape (Fig. 1a). Dynamic light scattering (DLS) analysis showed that the average size of ASC-MVs was 235.5 ± 15.4 nm (range 90–900 nm), and the size distribution by intensity was single-peaked, which confirmed the purity (100%) of isolated MVs (Fig. 1b). This infers that ASC-MVs were successfully isolated.

### ASC-MVs are internalized by HUVECs, HaCAT, and fibroblasts

To evaluate the internalization of ASC-MVs, cells including HUVECs, HaCAT, and fibroblasts were incubated with 20 μg PKH26-labeled MVs for 24 h. The red fluorescence was greatly observed in the cytoplasm of all three kinds of cells, which suggests that ASC-MVs could be readily internalized by these cells (Fig. 1c).

### ASC-MVs promote endothelial cell proliferation, migration, and angiogenesis

**Fig. 2:**
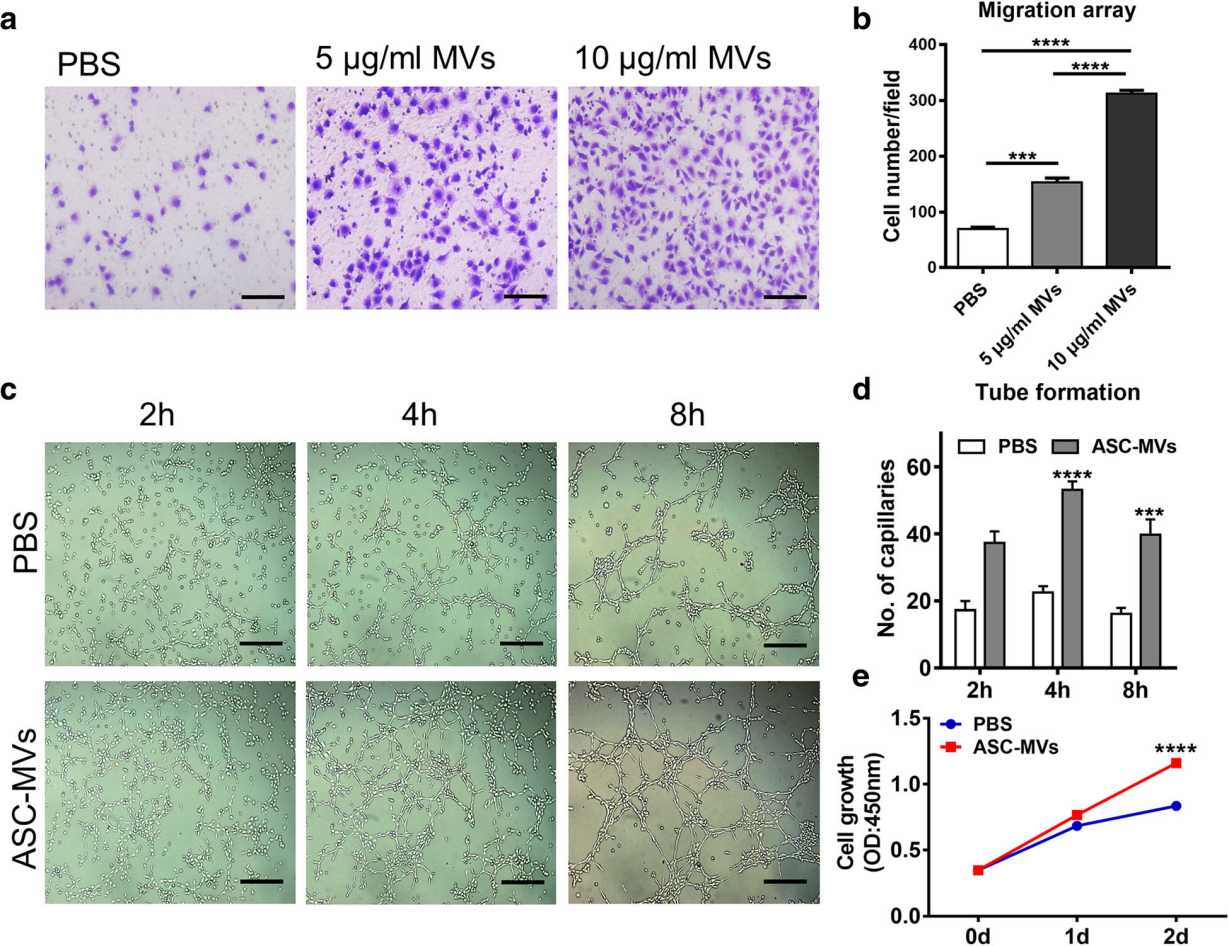
ASC-MVs induce HUVECs migration, proliferation, and angiogenesis. **a** Images of migrated HUVECs taken at 24 h after treatments of PBS, 5 μg/ml ASC-MVs, and 10 μg/ml ASC-MVs, respectively. Scar bar, 100 μm. **b** Qualification of migrated cells given different treatments (*n* = 3). **c** Enhanced tube formation in HUVECs treated with 20 μg/ml ASC-MVs compared with the controls at different time point. Scar bar, 50 μm. **d** Qualification of closed tubular structures shown in **c** (*n* = 3). **e** Increased HUVEC proliferation at day 2 after 20 μg/ml ASC-MV treatment compared with the controls as evidenced by CCK8 assay (*n* = 5). **p < 0.01, ***p < 0.001, ****p < 0.0001

To investigate whether ASC-MVs could promote the proliferation, migration, and angiogenesis of endothelial cells, we conducted a series of assays below. Firstly, HUVEC seeding in a Transwell system were treated with either 5 μg/ml ASC-MVs or 10 μg/ml ASC-MVs; significant differences were observed between the treated groups and untreated controls, with migration increasing 2.2-fold and 4.5-fold in 5 μg/ml ASC-MVs and 10 μg/ml ASC-MVs groups, respectively (Fig. 2a, b). Additionally, in vitro tube formation assay was performed to assess the ability of ASC-MVs to promote endothelial cell angiogenesis. As we expect, there was more than 2-fold increase of closed tubular structures in the treated group at 2 h, 4 h, and 8 h of incubation, which means ASC-MVs continuously promoted angiogenesis (Fig. 2c, d). Lastly, the treatment of 20 μg/ml ASC-MVs also greatly enhanced the endothelial cell proliferation as measured by CCK8 assay (Fig. 2e). Thus, these data suggest that ASC-MVs enhanced the HUVEC proliferation, migration, and angiogenesis in vitro.

### ASC-MVs modulate HaCAT and fibroblasts function in vitro

**Fig. 3:**
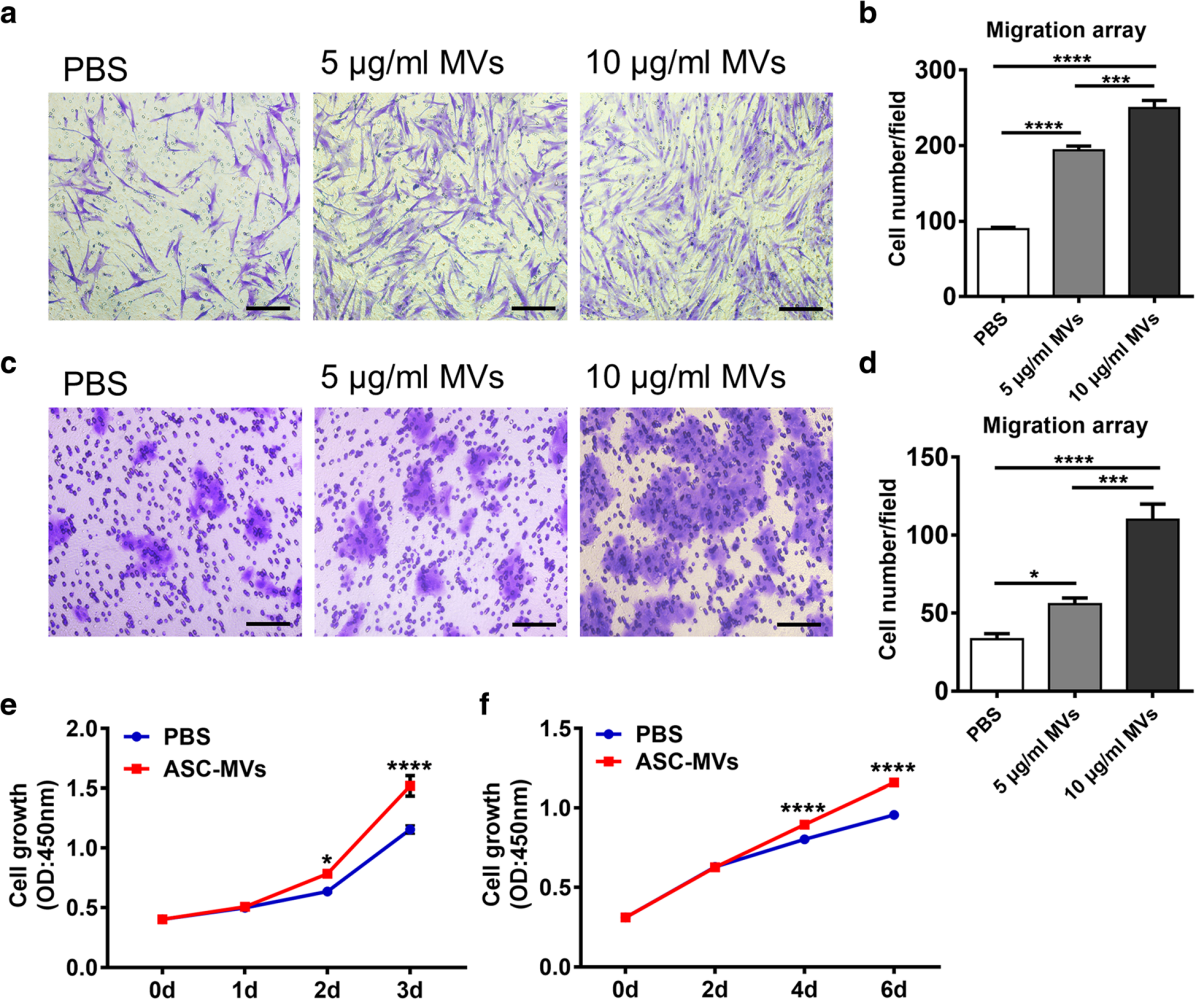
Modulation of HaCAT and fibroblast function by ASC-MV administration. **a** Images of migrated fibroblasts taken at 24 h after treatments of PBS, 5 μg/ml ASC-MVs, and 10 μg/ml ASC-MVs, respectively. Scar bar, 100 μm. **b** Qualification of data shown in **a** (*n* = 3). **c** Images of migrated HaCAT given the above treatment. Scar bar, 100 μm. **d** Qualification of data shown in **c** (*n* = 3). **e** Increased HaCAT proliferation at days 2 and 3 after 20 μg/ml ASC-MV treatment compared with the controls as evidenced by CCK8 assay (*n* = 5). **f** Increased fibroblast proliferation at days 4 and 6 after 20 μg/ml ASC-MV treatment (*n* = 6). *p < 0.05, **p < 0.01, ***p < 0.001, ****p < 0.0001

Keratinocytes and fibroblasts are the prime functional cells in the skin, so it is essential to evaluate whether ASC-MVs also enhance their cellular functions. ASC-MV treatment greatly promoted this cell migration: the number of migrated fibroblasts was increased 2.0-fold and 3.3-fold in 5 μg/ml ASC-MVs and 10 μg/ml ASC-MVs groups compared with untreated controls, respectively (Fig. 3a, b) and that was 2.2-fold and 2.8-fold in the case of HaCAT (Fig. 3c, d). Besides, in the CCK8 assay, the results showed that the proliferative ability of HaCAT and fibroblasts were augmented after 2 days and 4 days of treatment of ASC-MVs respectively, and even more significantly at 3 days and 6 days (Fig. 3e, f). Collectively, these dates indicate that ASC-MVs promote the proliferation and migration of HaCAT and fibroblasts in vitro.

### ASC-MVs upregulate genes associated with regenerative functions

**Fig. 4:**
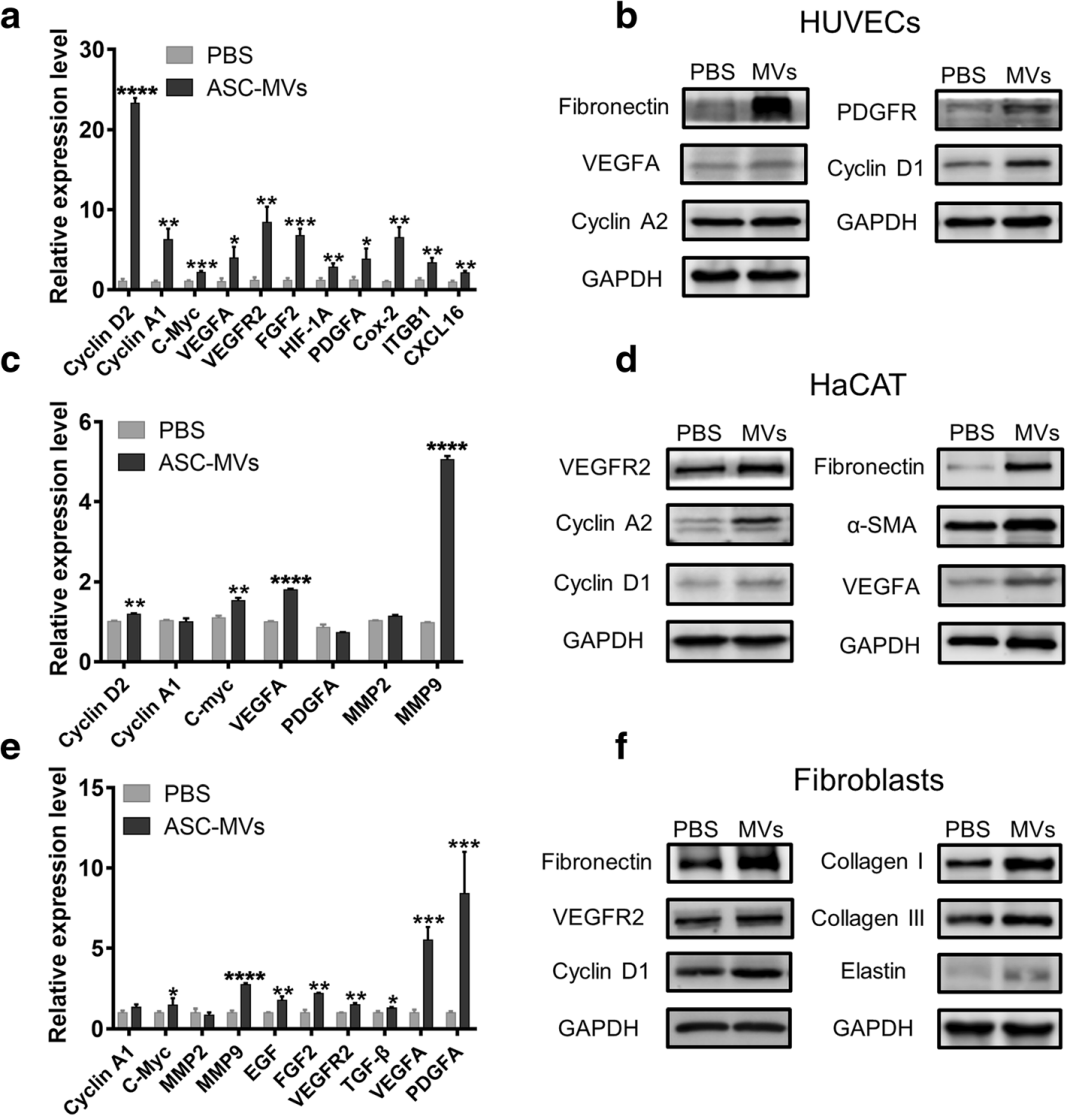
Effect of ASC-MV treatment on gene expression in all three kinds of cells. qRT-PCR analysis of a cluster of gene expression in cells treated with either PBS or 20 μg/ml ASC-MVs. **a** Upregulated genes in HUVECs. **c** Upregulated genes in HaCAT. **e** Upregulated genes in fibroblasts. Western blot analysis of gene expression in cells given above treatments. **b** Upregulated genes in HUVECs. **d** HaCAT. **f** Fibroblasts. GAPDH served as an internal control. *N* = 3. *p < 0.05, **p < 0.01, ***p < 0.001, ****p < 0.0001

Having confirmed that ASC-MV treatment augments the functions of three kinds of cells involved in skin repair, we also wondered whether this treatment could regulate the gene expression associated with regenerative phenotypes. The qRT-PCR analyses revealed that ASC-MV treatment profoundly upregulated cyclin D2 (23.2-fold), cyclin A1 (6.3-fold), c-Myc (2.2-fold), VEGFA (3.9-fold), VEGFR2 (8.4-fold), FGF2 (6.73-fold), HIF-1A (2.8-fold), PDGFA (3.8-fold), Cox-2 (6.5-fold), ITGB1 (3.4-fold), and CXCL16 (2.1-fold) in HUVECs (Fig. 4a). Furthermore, western blot analyses showed that cyclin D1, cyclin A2, VEGFA, PDGFR, and fibronectin were also upregulated after MV stimulation (Fig. 4b). Similarly, the mRNA expression levels of cyclin A1, c-Myc, VEGFA, and MMP9 were significantly increased after ASC-MV treatment in HaCAT, but there were no significant increases for cyclin A1, PDGFA, and MMP2 (Fig. 4c). And the enhanced expression levels of cyclin D1, cyclin A2, VEGFR, VEGFA, fibronectin, and α-SMA were further confirmed by western blot (Fig. 4d). As for fibroblasts, the results were similar: the qRT-PCR showed the levels of c-Myc, MMP9, EGF, FGF2, VEGFR, TGF-β, VEGFA, and PDGFA were increased 1.5-fold, 2.8-fold, 1.8-fold, 2.2-fold, 1.5-fold, 1.3-fold, 5.5-fold, and 8.4-fold after MV stimulation, respectively (Fig. 4e); the levels of VEGFR2, cyclin D1, fibronectin, collagen I, collagen III, and elastin were also enhanced as evidenced by western blot (Fig. 4f). In conclusion, ASC-MV treatment upregulated genes associated with the proliferation, migration, and angiogenesis of HUVECs, HaCAT, and fibroblasts.

### ASC-MVs promote the activation of AKT and ERK signaling pathways

**Fig. 5:**
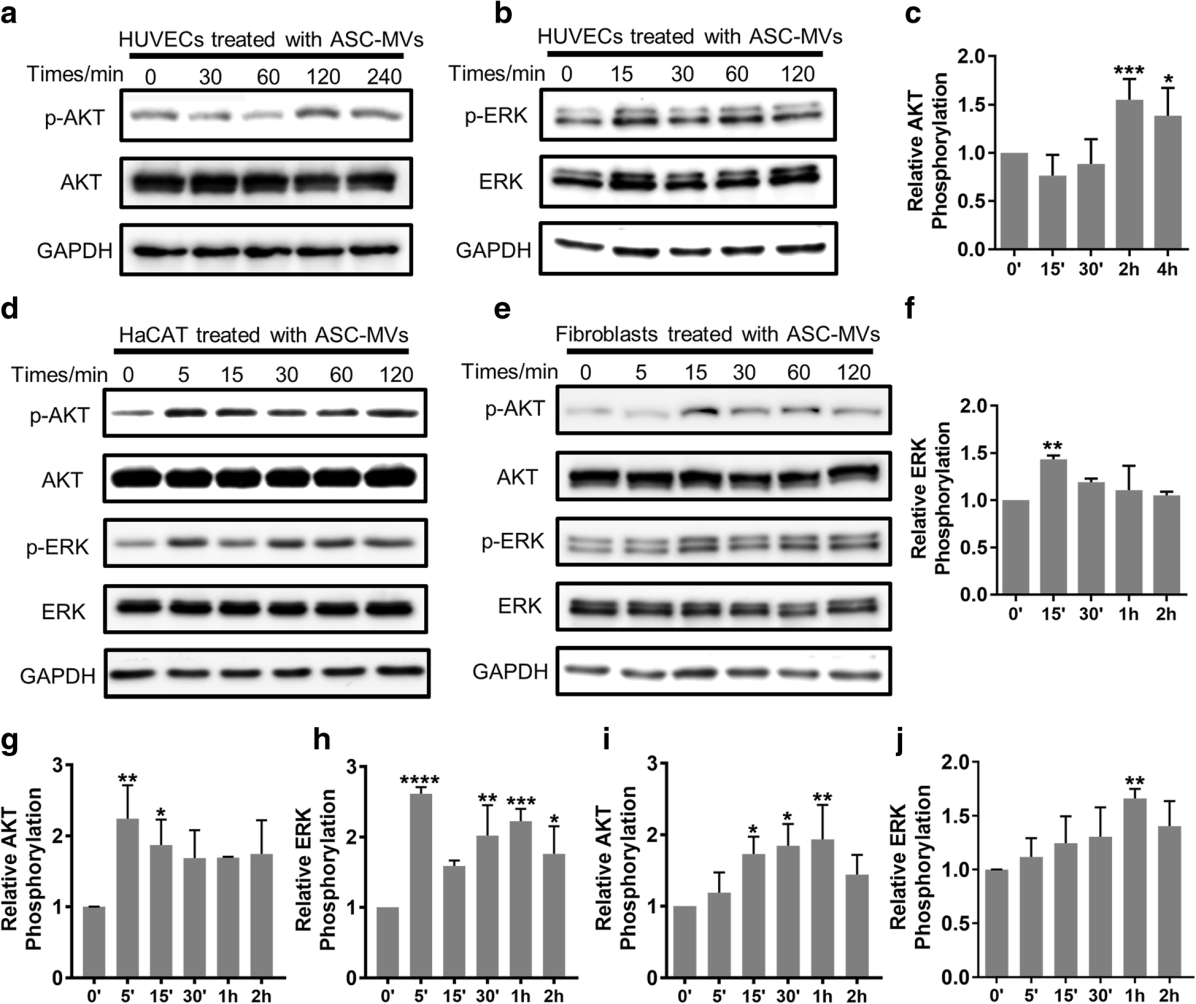
Effect of ASC-MVs on the activation level of AKT and ERK signaling pathways. Western blot analysis of the phosphorylation level of AKT and ERK1/2 in cells treated with 20 μg/ml ASC-MVs for the indicated lengths of time. **a** The ratio of p-AKT/AKT in HUVECs was examined and included on the blots at different time points. **b** The ratio of p-ERK/ERK in HUVECs was determined. **d** The ratio of p-ERK/ERK and p-AKT/AKT in HaCAT was determined. **e** The ratio of p-ERK/ERK and p-AKT/AKT in fibroblasts was determined. Qualification of the ratio of p-ERK/ERK and p-AKT/AKT at different time points. **c**, **f** Qualified data shown in **a** and **b**. **g**, **h** Qualified data shown in **d**. **i**, **j** Qualified data shown in **e**. *N* = 3. *p < 0.05, **p < 0.01, ***p < 0.001, ****p < 0.0001

Next, we sought to investigate which signaling pathways were involved in the regenerative effects of ASC-MVs. We used DAVID database to analyze the genes upregulated by ASC-MVs. The results revealed that 11, 8, 7, and 4 of all 18 upregulated genes were involved in PI3K-AKT, ERK, Rap1, and FoxO signaling pathways, respectively (Additional file 3: Figure S2). Therefore, PI3K-AKT and ERK signaling pathways were of the first concerned. Indeed, we found that ASC-MVs induced a time-dependent phosphorylation of AKT (p-AKT) and ERK1/2 (p-ERK1/2) in all three kinds of cells. In HUVECs, the phosphorylation of ERK1/2 and AKT was elevated maximally after 15 min and 2 h of MV exposure respectively (Fig. 5a–c, f). In HaCAT, the phosphorylation of ERK1/2 and AKT was maximally elevated 2.6-fold and 2.2-fold at 5 min and remained elevated until the last observed time point (Fig. 5d, g, h). As for fibroblasts, the phosphorylation of ERK1/2 and AKT was elevated gradually since 5 min, maximal after 1 h of MVs exposure, and decreasing at 2 h (Fig. 5e, i, j). Therefore, the activation level of AKT and ERK signaling pathways in all three kinds of cells was augmented by ASC-MVs.

### ASC-MV transplantation accelerates cutaneous wound healing in mice

**Fig. 6:**
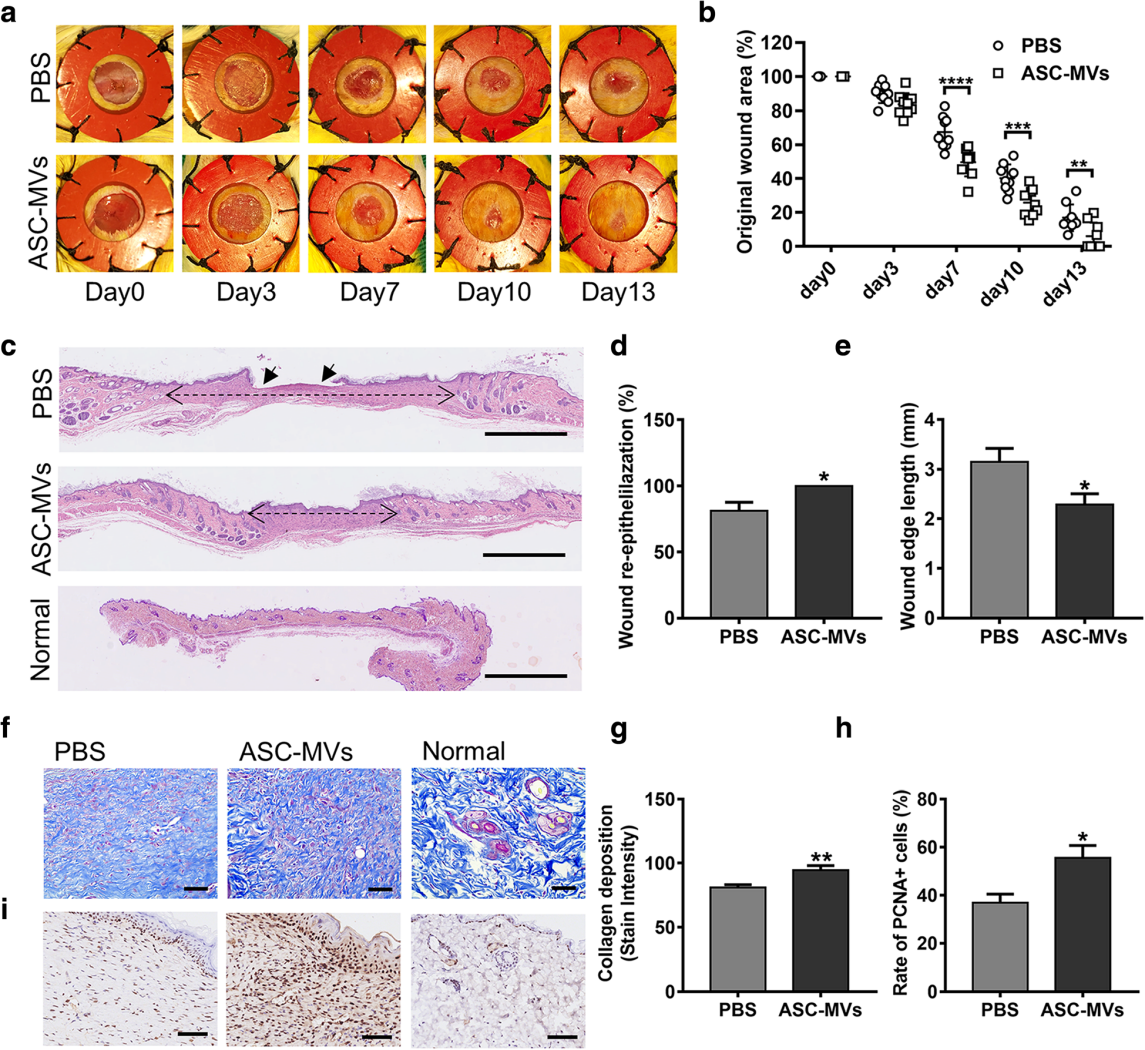
Effect of ASC-MVs on wound healing in mice. **a** Gross view of excisional wounds in mice treated with either 50 μg ASC-MVs or an equal volume of PBS at different time points (*n* = 9). **b** Measurement of wound areas shown in **a**. **c** H&E staining analysis of wound sections following different treatments at day 13 post-wounding. The single-headed arrows indicate the un-epithelialized areas. The double-headed arrows indicate the edges of the granulation. Scar bar, 1 mm. **d** Qualification of wound re-epithelialization shown in **c**. **e** Qualification of granulation tissue formation shown in **c**. **f** Evaluation of collagen deposition by Masson staining at day 13 post-wounding. **g** Qualification of the stain intensity of blue collagen shown in **f**. **h** Qualification of the ratio of PCNA+ cells in wound beds. **i** Immunohistochemical staining for PCNA expression in wound sections. Scar bar, 100 μm. *p < 0.05, **p < 0.01, ***p < 0.001, ****p < 0.0001

To evaluate the therapeutic potential of ASC-MVs on wound healing, full-thickness wounds were created at the dorsum of mice, followed by the local injection of 50 μg ASC-MVs (*n* = 9/group) or an equal volume of MV diluent (PBS) around the wound area. The results showed that the original wound area in mice treated with MVs was significantly smaller compared to controls at days 7, 10, and 13 post-wounding (Fig. 6a, b). Even more striking, the wounds in five mice treated with MVs were completely healed at day 13 post-wounding but none of the control mice. Additionally, the in vivo tracking experiment showed that ASC-MVs could exist 15 days in the wound area, which was consistent with the time until complete wound closure (Additional file 4: Figure S3).

Next, histological analysis was performed to assess the degree of wound healing and regeneration. H&E staining analysis showed that wound re-epithelialization in MV-treated group was remarkably enhanced, and wound edges were significantly narrowed at day 13 post-wounding (Fig. 6c–e). Moreover, a larger and better-organized collagen deposition was observed in the wounds of mice treated with MVs (Fig. 6f, g). These data indicate that ASC-MVs significantly accelerated wound re-epithelialization and collagen deposition and thus promoted wound healing.

### ASC-MVs augment neovascularization and cellular proliferation in vivo

**Fig. 7:**
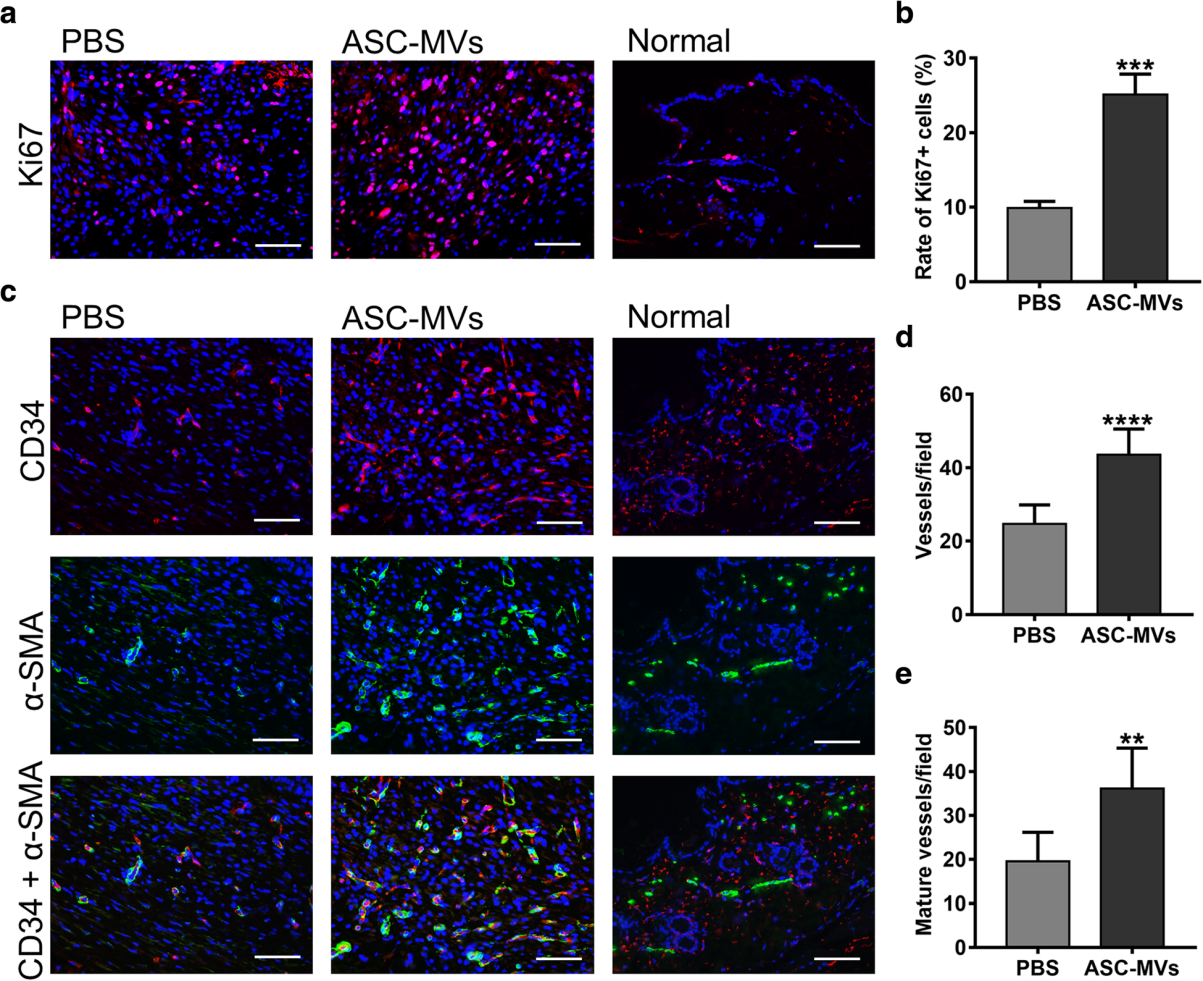
ASC-MVs enhanced neovascularization and cellular proliferation in vivo as evidenced by immunofluorescence analysis. **a** Ki-67 (red color) immunofluorescence staining of wound sections treated with either 50 μg ASC-MVs or an equal volume of PBS at day 13 post-wounding. **b** Qualification of the ratio of Ki67+ cells in wound beds. **c** Immunofluorescent triple staining of wound sections given the above treatments at day 13 post-wounding. Smooth muscle cells (a-SMA), endothelial cells (CD34), and cell nuclei (DAPI) were stained with green, red, and blue colors. Newly formed vessels express CD34. Mature vessels express both CD34 and a-SMA. **d** Enumeration of newly formed vessels stained with red color. **e** Enumeration of mature vessels co-stained with red and green colors. Scar bar, 100 μm. *N* = 7. **p < 0.01, ***p < 0.001, ****p < 0.0001

Immunohistochemical analysis of wounds was carried out to investigate whether ASC-MVs induce morphometric changes in the skin. Firstly, the cellular proliferation ability in the MV-treated group was markedly enhanced as confirmed by the increased rate of PCNA+ and Ki67+ cells (Fig. 6h, i and Fig. 7a, b). Next, capillary density was significantly increased in the wound areas treated with MVs as evidenced by CD34 staining (Fig. 7c, d). Additionally, CD34 and α-SMA co-staining analysis revealed that the number of mature vessels in the MV-treated group was also significantly increased (Fig. 7c, e). Collectively, these results indicate that ASC-MVs promoted cellular proliferation and neovascularization in vivo, which are the two prime processes of wound healing.

## Discussion

Although originally considered as waste carriers, extracellular vesicles comprising exosomes and microvesicles are now comprehended to play an important role in intercellular communication [25]. Recently, studies shed light on the fundamental research and translational application of exosomes in tumorigenesis and tissue repair [26, 27]. However, less effort has been focused on microvesicles. In the present study, we investigated whether ASC-MVs have therapeutic effects in a mouse excisional wound healing model and explored the underlying mechanisms involved. Assessing the effects of ASC-MVs on key cell types involved in wound healing, we found that ASC-MVs can promote cell proliferation, migration, and vascularization both in vitro and in vivo; upregulate a number of genes modulating cellular function; lead to the activation of AKT and ERK signaling pathways; and thus accelerate wound healing and regeneration.

Stem cell therapies for tissue repair have been overwhelming and extensively studied for the past decades. However, apart from significant benefits of hematopoietic stem cell therapy on hematological diseases, limited successes have been achieved in clinical application because of lacking sufficient evidences on safety and efficacy [9]. Interestingly, it has been well-documented that the paracrine factors of stem cells contribute mainly to the therapeutic effect, which includes growth factors, cytokines, and extracellular vesicles [26]. Recently, studies have demonstrated that extracellular vesicles are involved in the transfer of biological molecules between cells. This effect has the potential to be harnessed for wound healing without the risks of immunorejection and teratomas. Despite of abundant resources of stem cells in the human body, adipose stem cells can act as an ideal origin of extracellular vesicles owing to their ease of harvest, fast cultural expansion, and low immunogenic features. Previous works have paid more attention on the effect of exosomes isolated from various kinds of stem cells on wound healing [13]. Here, we demonstrate that microvesicles have a therapeutic capacity for wound healing when isolated from adipose stem cells by the classical differential ultracentrifugation method. Moreover, our study found that ASC-MVs could be easily internalized by HUVECs, HaCAT, and fibroblasts, which suggests that ASC-MVs can act as appropriate vehicles in the transport of various biomolecules and signals to these cells.

The process of wound repair can be divided into three overlapping steps: inflammation, new tissue formation, and remodeling [3]. The second step is characterized by proliferation, migration, and angiogenesis of different cell types (mainly keratinocytes, fibroblasts, and endothelial cells) [3, 28]. Therefore, we evaluated the effect of ASC-MVs on cells proliferation. Previous works showed that exosomes isolated from human iPSC-derived MSCs, ASCs, and human umbilical cord can promote the proliferation of human fibroblasts [17, 20, 29]. Zhang et al. reported that exosomes derived from human EPCs enhance the HMEC proliferation and increase cyclin D1 and c-Myc gene expression [30], which are well-known proliferation-associated genes. In the present study, we found that ASC-MVs can promote the proliferation of HUVECs, HaCAT, and fibroblasts both in vitro and in vivo as evidenced by CCK8 assay and immunohistochemical staining of PCNA and Ki67. Furthermore, this phenomenon has been confirmed by increased gene expression of cyclin D1, cyclin D2, cyclin A1, and cyclin A2, which are involved in the cell cycle and regarded as proliferative markers [31]. Besides, the upregulation of c-Myc may partially account for the effect of ASC-MVs on cell proliferation, because this gene can accelerate cell cycle progression by many pathways including targeting CDKs and cyclins [32].

The migration ability of HUVECs, HaCAT, and fibroblasts was also enhanced by ASC-MVs, which was consistent with other reports [20, 21]. Importantly, fibronectin, which is involved in the cell adhesion and migration processes, was significantly upregulated in all three kinds of cells. This finding resembles other research, which illustrated the important role of fibronectin in the involvement of MVs from embryonic stem cells promoting trophoblasts migration and embryo implantation [33]. Previous work showed that platelet microvesicles promote the migration and angiogenesis of HUVECs by upregulating MMP2 and MMP9 expression [22]. In this study, we found that the expression level of MMP9 was markedly increased in HaCAT and fibroblasts by ASC-MV stimulation, which may contribute to the enhanced migration ability of these cells despite of no increase in MMP2 expression. Additionally, we found that ASC-MVs significantly upregulated the gene expression of integrin beta 1 (ITGB1) and CXCL16 in HUVECs, which can also modulate cell migration as reported previously [34, 35].

Impaired wound healing is often associated with abnormalities of blood supply in wound beds. Previous studies have demonstrated that exosomes isolated from various cell types could promote angiogenesis and neovascularization [16, 17, 20, 30]. In our study, we found that ASC-MVs significantly increase the number of closed tubular structures in vitro. Similarly, an in vivo assay also showed ASC-MVs could increase the number of newly formed vessels and mature vessels. These results suggest that ASC-MVs can improve blood supply in wound beds. Moreover, a number of growth factors and receptors including PDGFA, VEGFA, FGF2, HIF-1A, VEGFR2, and PDGFR were significantly upregulated by ASC-MVs in HUVECs, which may explain the angiogenesis effect of ASC-MVs on endothelial cells. Meanwhile, we found that ASC-MVs could also increase the levels of PDGFA, VEGFA, FGF2, EGF, and VEGFR2 in HaCAT or fibroblasts. This may contribute to the neovascularization of endothelial cells in wounds by paracrine pathways.

Having confirmed that ASC-MVs could modulate cellular function and regulate related genes, we next explored the involvement of signaling pathways. AKT, ERK, STAT3, and WNT signaling pathways are recognized to play important roles in wound healing [3, 36]. Gangadaran et al. reported that the activation of AKT and ERK signaling pathways by extracellular vesicles from MSC contribute to the proliferation, migration, and tube formation of endothelial cells [37]. Zhang et al. showed that exosomes from ASCs promote fibroblasts proliferation and collagen secretion via PI3K/AKT signaling pathway [20]. Human umbilical cord MSC-derived exosomes could also enhance HaCAT proliferation and migration via activating WNT/β-catenin signaling pathway [18]. In order to accurately find the underlying signaling pathways, we analyzed the upregulated genes by DAVID database [38, 39]. The results showed that most of these genes are the members of AKT and ERK signaling pathways. Combining the results in our previous studies, AKT and ERK signaling pathways are most likely to be involved in the regenerative effects of ASC-MVs. Not surprisingly, we found that ASC-MVs could augment the activation level of AKT and ERK signaling pathways in HUVECs, HaCAT, and fibroblasts. Thus, these two signaling pathways may contribute to the therapeutic effect of ASC-MVs on wound healing. Further studies should be done to assess whether inhibition of AKT and ERK signaling pathways impairs the capacity of ASC-MVs on cellular function and wound repair.

Finally, we observed that ASC-MV transplantation significantly accelerated wound closure and re-epithelialization in an established wound model that eliminates the interference of wound contraction [40]. We also found that ASC-MVs greatly increased the collagen deposition in wound beds, and the result was consistent with our in vitro study, which showed that the expression levels of collagen I, collagen III, and elastin were increased in fibroblasts after ASC-MV treatment. Meanwhile, the distance between wound edges, which is often referred to as scars in previous works [17, 20], was narrowed after ASC-MV treatment. Moreover, an in vivo tracking experiment showed that the lifespan of ASC-MVs in murine wounds was 15 days, which mainly comprised of inflammation and new tissue formation stages of the wound healing process. Since the remodeling process often starts at 2–3 weeks after injury and can last for 1 year or more [3], ASC-MVs may not exist during that stage. However, it is necessary to conduct a longer observation in further studies to investigate the later effect of ASC-MVs on scar formation.

## Conclusion

In this study, we demonstrated that ASC-MVs could promote re-epithelialization, collagen deposition, and neovascularization at wound sites and thus accelerate wound closure in mice. The therapeutic capacity of ASC-MVs may be due to their effect on proliferation, migration, and angiogenesis of keratinocytes, fibroblasts, and endothelial cells. The underlying mechanisms may be the activation of AKT and ERK signaling pathways and the increased expression of various growth factors, cyclins, and other genes in cells after ASC-MV stimulation. Thus, our findings suggest that the application of ASC-MVs may represent a promising therapeutic strategy for improving cutaneous wound healing in patients.

## Additional files

Additional file 1: **Table S1.** Primers used for real-time polymerase chain reaction. (DOCX 14 kb)

Additional file 2: **Figure S1.** Characterization of ASC. **a** Flow cytometry revealed that more than 90% ASCs highly expressed CD73, CD90, and CD44 but less than 3% ASCs expressed CD34 and HLA-DR. **b** ASCs were adherent and fibroblast-like cells. **c** Adipogenic differentiation assay showed that ASCs could differentiate into adipocytes as stained with Oil Red O. **d** Osteogenic differentiation assay showed that ASCs could differentiate into osteocytes as evidenced by Alizarin Red staining. (TIF 2146 kb)

Additional file 3: **Figure S2.** KEGG pathway analysis of genes upregulated by ASC-MVs. This image listed the four most relevant signaling pathways associated with 18 upregulated genes as analyzed by DAVID database. (TIF 1032 kb)

Additional file 4: **Figure S3.** In vivo tracking of subcutaneously injected ASC-MVs. **a** Representative fluorescence imaging of mice wounds treated with 50 μg PKH26-labeled ASC-MVs, PKH26, or PBS was detected at indicated time points. **b** The fluorescence net intensity was used to assess the residual content of PKH26-labeled ASC-MVs or PKH26 in mice. More than 95% of fluorescence net intensity in PKH26 injected mice was eliminated at day 10, and no fluorescence was detected at day 15. More than 95% of fluorescence net intensity in PKH26-labeled ASC-MVs injected mice was eliminated at day 15. No fluorescence was detected in PBS injected mice. *N* = 3. (TIF 803 kb)

## Abbreviations

AKT: AKT serine/threonine kinase 1
ASC-MVs: Adipose stem cell-derived microvesicles
ASCs: Adipose stem cells
BSA: Bull serum albumin
CCK8: Cell Counting Kit-8
c-Myc: MYC proto-oncogene
Cox-2: Prostaglandin-endoperoxide synthase 2
CXCL16: C-X-C motif chemokine ligand 16
DLS: Dynamic light scattering
EGF: Epidermal growth factor
EPCs: Endothelial progenitor cells
ERK: Mitogen-activated protein kinase 1
FGF2: Fibroblast growth factor 2
GAPDH: Glyceraldehyde-3-phosphate dehydrogenase
HIF-1A: Hypoxia-inducible factor 1 subunit alpha
hiPSC-MSCs: Human induced pluripotent stem cell-derived MSCs
huMSCs: Human umbilical cord MSCs
HUVECs: Human umbilical vein endothelial cells
ITGB1: Integrin subunit beta 1
Ki-67: Antigen Ki-67
MMP2: Matrix metallopeptidase 2
MMP9: Matrix metallopeptidase 9
MSCs: Mesenchymal stem cells
PBS: Phosphate-buffered saline
PCNA: Proliferating cell nuclear antigen
PDGFA: Platelet-derived growth factor subunit A
PDGFR: Platelet-derived growth factor receptor beta
PSCs: Pluripotent stem cells
qRT-PCR: Quantitative reverse-transcriptase polymerase chain reaction
TGF-β: Transforming growth factor beta 1
VEGFA: Vascular endothelial growth factor A
VEGFR2: Kinase insert domain receptor
α-SMA: Actin, alpha 2, smooth muscle, aorta
