# Supplementary material for: Microvesicles from human adipose stem cells promote wound healing by optimizing cellular functions via AKT and ERK signaling pathways
Source: Stem Cell Res Ther. 2019 Jan 31;10:47. doi: 10.1186/s13287-019-1152-x (PMC6357421; doi:10.1186/s13287-019-1152-x)
Supplement: Supplementary file 1 — Table S1. Primers used for real-time polymerase chain reaction. (DOCX 14 kb) [file 13287_2019_1152_MOESM1_ESM.docx]

**Table S1** Primers used for real-time polymerase chain reaction

| Gene | Primer sequences |
| --- | --- |
| Cyclin D2  Cyclin A1  c-Myc  VEGFA  VEGFR2  FGF2  HIF-1A  PDGFA  Cox-2  ITGB1  CXCL16  EGF  TGF-β  MMP2  MMP9  GAPDH | Forward 5’-GAAGTGTGGGAGCAGCCATCT-3’  Reverse 5’-CACGTTGGTCCTGACGGTACT-3’  Forward 5’-GGCTGCTAACTGCAAATGGGC-3’  Reverse 5’-ATGTCAAACCCTTGCTTGGGG-3’  Forward 5’-CCTTCTCTCCGTCCTCGGATT-3’  Reverse 5’-ACTCTGACCTTTTGCCAGGAGC-3’  Forward 5’-AAGGCGAGGCAGCTTGAGTT-3  Reverse 5’-CGGCAGCGTGGTTTCTGTATC-3  Forward 5’-AACCGGAACCTCACTATCCGC-3  Reverse 5’-TTCGTCTTTTCCTGGGCACCT-3  Forward 5’-GAGAAGAGCGACCCTCACATCA-3  Reverse 5’-TGCCCAGTTCGTTTCAGTGCC-3  Forward 5’-GCCTTGGATGGTTTTGTTATGGT-3  Reverse 5’-AGCTTCGCTGTGTGTTTTGTT-3  Forward 5’-GGATACCTCGCCCATGTTCTG-3  Reverse 5’-GCTTCCTCGATGCTTCTCTTCC-3  Forward 5’-AAATTGCTGGCAGGGTTGCTGG-3  Reverse 5’-AAGGGCAGGATACAGCTCCACA-3  Forward 5’-TGGAATTGTTCTTATTGGCCTTGC-3  Reverse 5’-TAGAGACCAGCTTTACGTCCG-3  Forward 5’- GTCTATACTACACGAGGTTCCAGC-3  Reverse 5’-AGTGGACTGCAAGGTGGACAG-3  Forward 5’-TGGATGGTTCAAAACGCCGAAG-3  Reverse 5’-ACGTACTCTATCTTTGCCAGTCCT-3  Forward 5’-AGGGCTACCATGCCAACTTCT-3  Reverse 5’-AGTACACGATGGGCAGCGG-3  Forward 5’-CCGGTTCATTTGGCGGACTG-3  Reverse 5’-CAGGGTGCTGGCTGAGTAGAT-3  Forward 5’-TGCCTTTGGACACGCACGAC-3  Reverse 5’-GTATCCGGCAAACTGGCTCCT-3  Forward 5’-AAAATCAAGTGGGGCGATGCT-3  Reverse 5’-TGGTTCACACCCATGACGAAC-3 |
