# Supplementary figures and images for: Microvesicles from human adipose stem cells promote wound healing by optimizing cellular functions via AKT and ERK signaling pathways
Source: Stem Cell Res Ther. 2019 Jan 31;10:47. doi: 10.1186/s13287-019-1152-x (PMC6357421; doi:10.1186/s13287-019-1152-x)

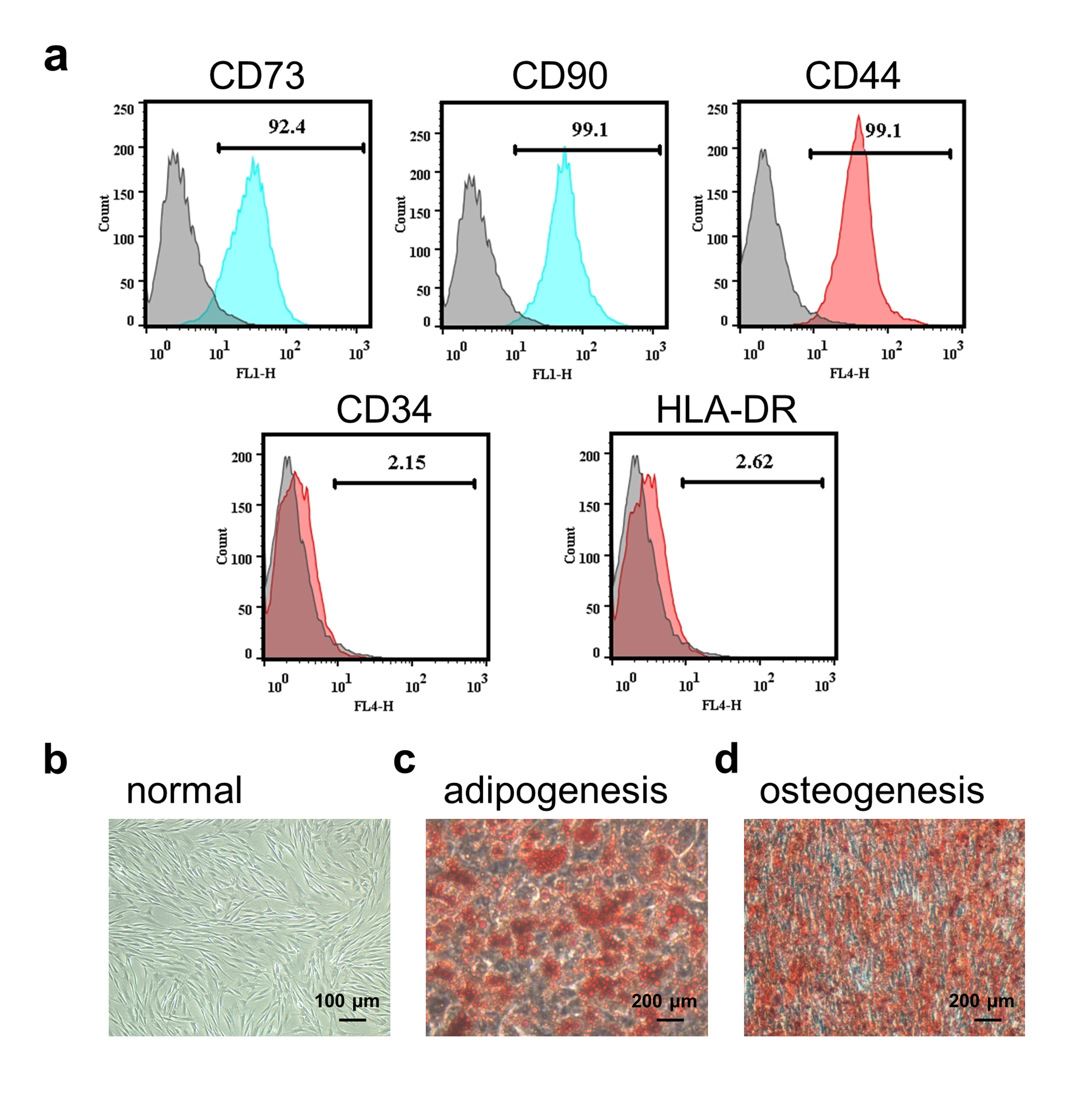

Supplement: Supplementary file 2 — Figure S1. Characterization of ASC. a Flow cytometry revealed that more than 90% ASCs highly expressed CD73, CD90, and CD44 but less than 3% ASCs expressed CD34 and HLA-DR. b ASCs were adherent and fibroblast-like cells. c Adipogenic differentiation assay showed that ASCs could differentiate into adipocytes as stained with Oil Red O. d Osteogenic differentiation assay showed that ASCs could differentiate into osteocytes as evidenced by Alizarin Red staining. (TIF 2146 kb) [file 13287_2019_1152_MOESM2_ESM.tif]

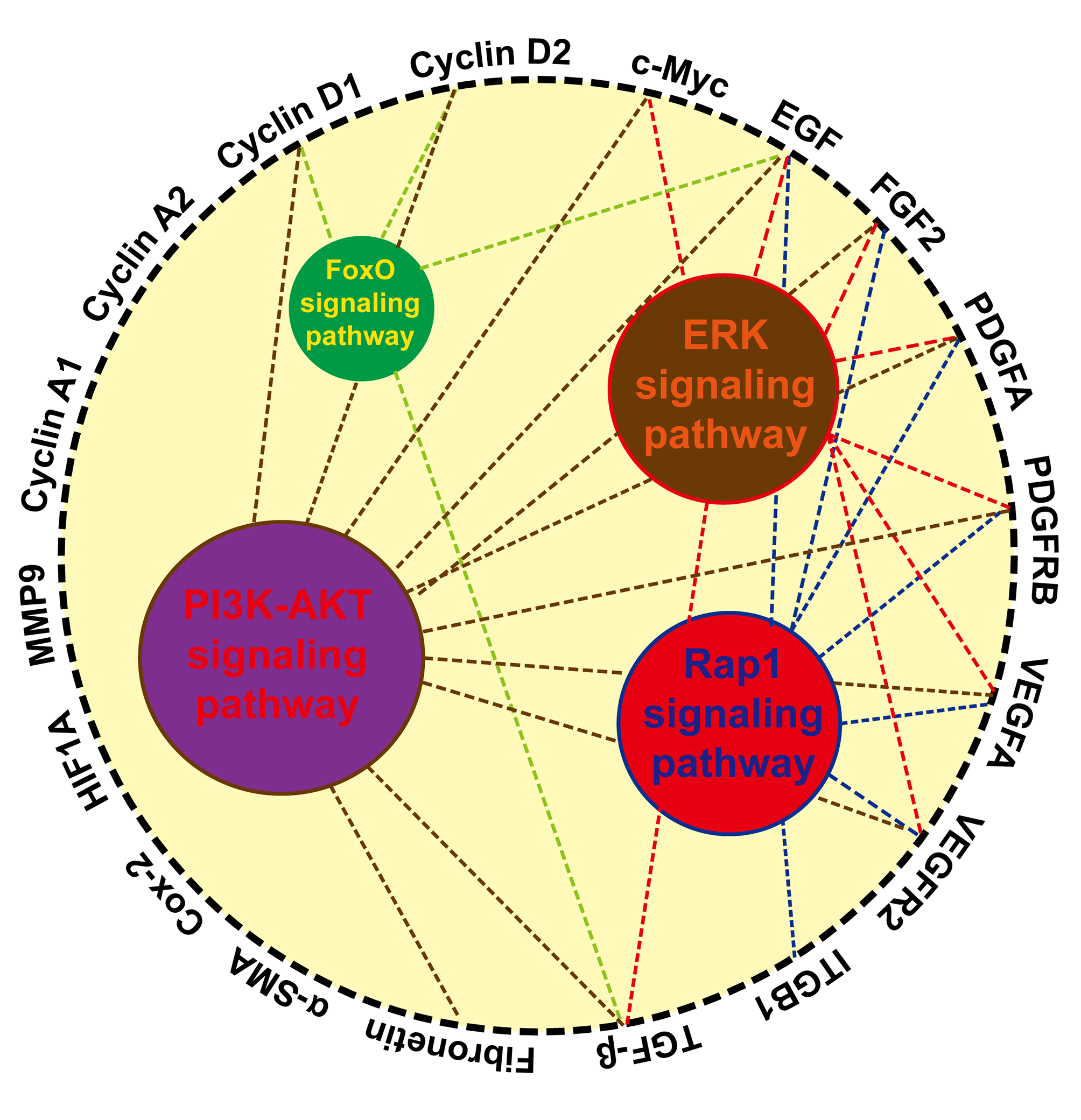

Supplement: Supplementary file 3 — Figure S2. KEGG pathway analysis of genes upregulated by ASC-MVs. This image listed the four most relevant signaling pathways associated with 18 upregulated genes as analyzed by DAVID database. (TIF 1032 kb) [file 13287_2019_1152_MOESM3_ESM.tif]

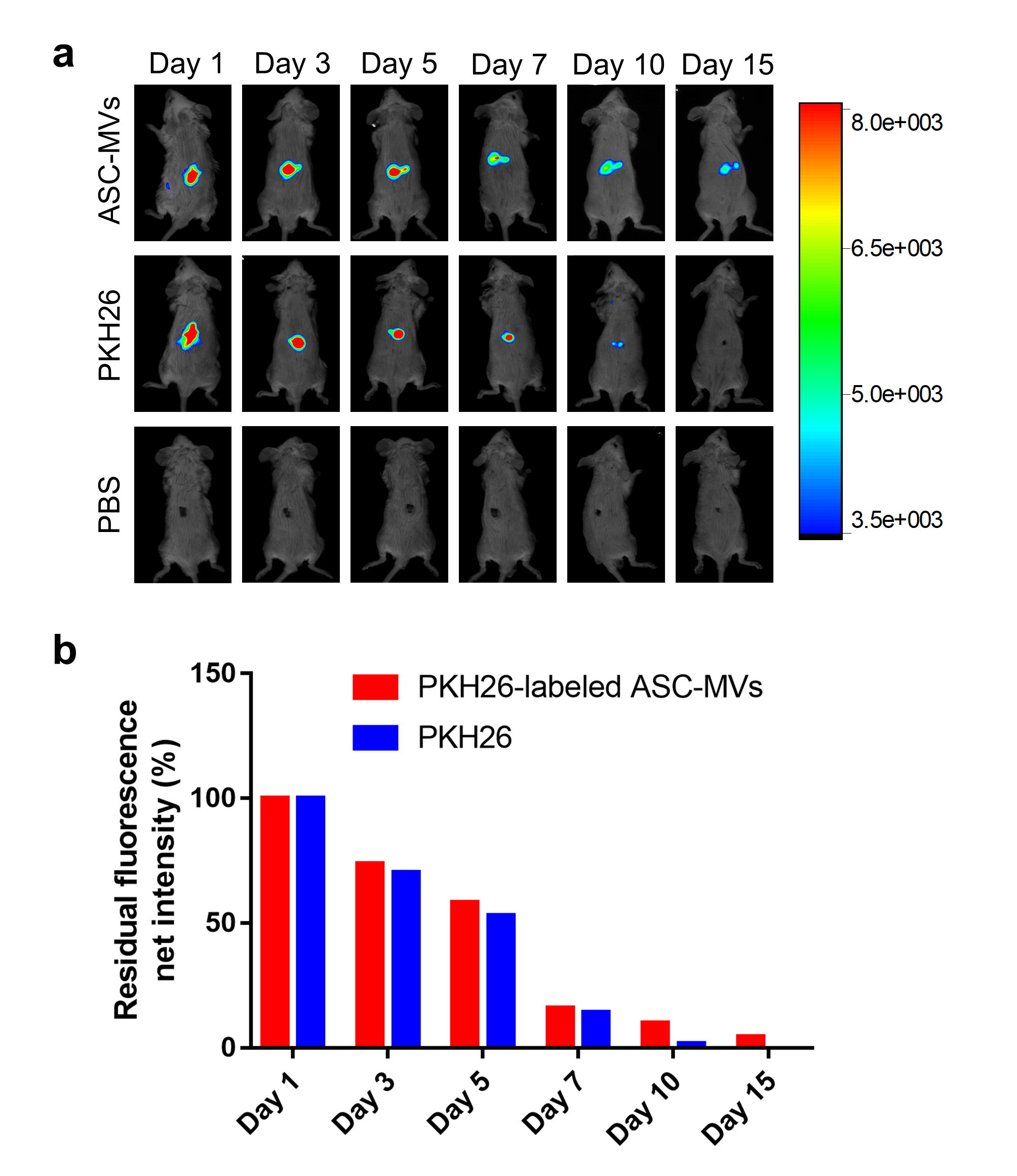

Supplement: Supplementary file 4 — Figure S3. In vivo tracking of subcutaneously injected ASC-MVs. a Representative fluorescence imaging of mice wounds treated with 50 μg PKH26-labeled ASC-MVs, PKH26, or PBS was detected at indicated time points. b The fluorescence net intensity was used to assess the residual content of PKH26-labeled ASC-MVs or PKH26 in mice. More than 95% of fluorescence net intensity in PKH26 injected mice was eliminated at day 10, and no fluorescence was detected at day 15. More than 95% of fluorescence net intensity in PKH26-labeled ASC-MVs injected mice was eliminated at day 15. No fluorescence was detected in PBS injected mice. N = 3. (TIF 803 kb) [file 13287_2019_1152_MOESM4_ESM.tif]
